# Tip of an Iceberg: Skull Fracture as an Adult Presentation of Encephalocraniocutaneous Lipomatosis

**DOI:** 10.1155/2016/3292654

**Published:** 2016-11-02

**Authors:** Sinead Culleton, Christen D. Barras, Hamed Asadi, Seamus Looby, Paul Brennan, Hong Kuan Kok

**Affiliations:** ^1^Neuroradiology and Neurointerventional Service, Department of Radiology, Beaumont Hospital, Dublin 9, Ireland; ^2^Lysholm Department of Neuroradiology, The National Hospital for Neurology and Neurosurgery, Queen Square, London, UK; ^3^Department of Radiology, The University of Melbourne, Parkville, VIC 3050, Australia; ^4^School of Medicine, Faculty of Health, Deakin University, Pigdons Road, Waurn Ponds, VIC 3216, Australia

## Abstract

The severity of seizures presenting to the emergency department ranges from benign to life threatening. There are also a wide number of possible etiologies. Computed tomography (CT) emergency imaging may be required at presentation to elucidate a possible cause and assess signs of intracranial trauma. This case describes a serious seizure episode in a young man while on holiday. A CT brain showed a skull fracture as a consequence of seizure-related head trauma but unexpectedly there were image findings consistent with encephalocraniocutaneous lipomatosis. The important radiological features of encephalocraniocutaneous lipomatosis and a differential diagnosis are presented.

## 1. Case Report

A 26-year-old male overseas visitor presented with head trauma and reduced consciousness with a Glasgow coma scale (GCS) of 3 following a prolonged tonic-clonic seizure which resulted in head trauma. He had a past history of developmental delay and epilepsy. No prior imaging was available for comparison.

**Figure 1:**
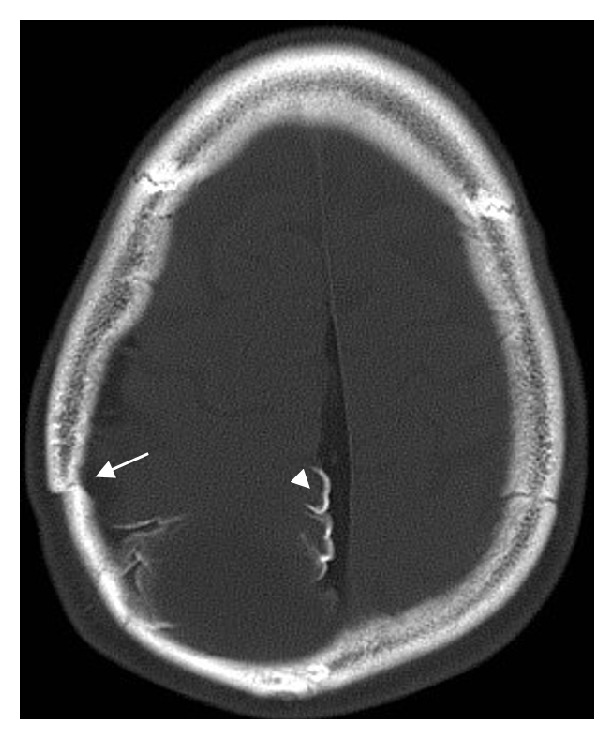
Noncontrast CT bone windows. Fracture of right parietal bone (arrow). Cortical gyriform calcifications are seen (arrowhead).

**Figure 2:**
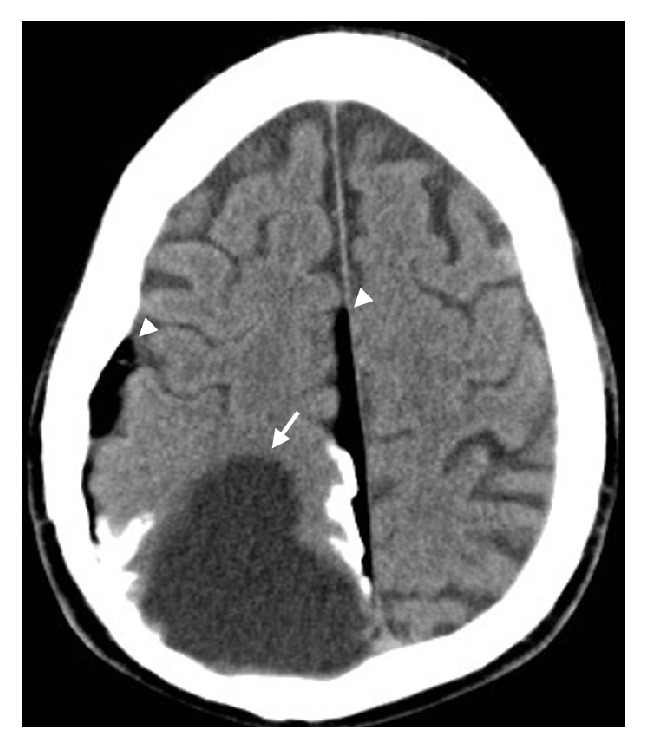
Noncontrast CT brain with a large right posterior parietal porencephalic cyst with adjacent cortical calcification (arrow). There is intracranial fat tracking along the falx and subjacent to the right parietal bone (arrowheads).

**Figure 3:**
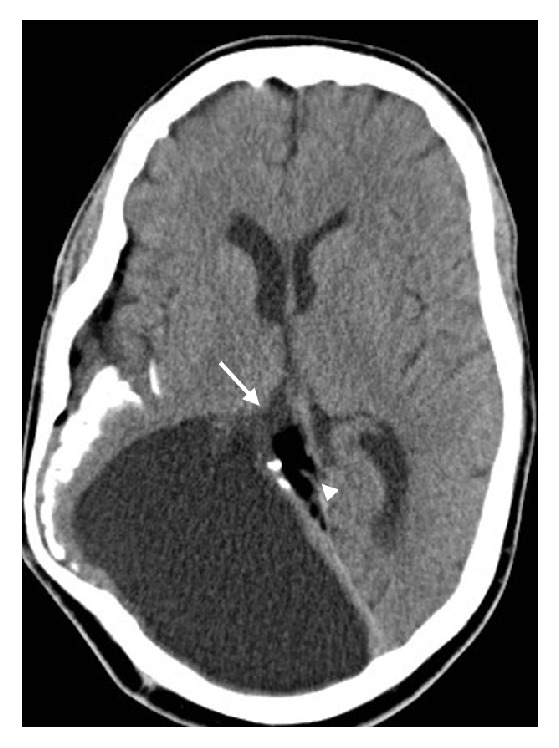
The porencephalic cyst directly communicates with the posterior horn of the right lateral ventricle (arrow) with adjacent cerebral lipomatosis (arrowhead) and cerebral atrophy.

A noncontrast CT brain showed a minimally depressed right parietal bone fracture (Figure 1) but no associated intracranial haemorrhage. A right parietooccipital porencephalic cyst was located subjacent to the fracture, with mild dilation of the right lateral ventricle and hemicerebral atrophy (Figure 2). There was associated right calvarial remodelling, localised cerebral cortex calcification, and surrounding multifocal lipomatosis (Figures 2 and 3). The left cerebral hemisphere was normal.

On clinical examination, there was alopecia involving his right scalp and an overlying subcutaneous soft tissue mass. The clinical and radiological findings were consistent with a diagnosis of encephalocraniocutaneous lipomatosis (ECCL), also known as Haberland syndrome. In this case, the individual met a number of diagnostic criteria for ECCL proposed by Moog et al. (Table 1 and Diagnosis Based on Minor and Major Criteria). These included two major criteria of a naevus psiloliparus and intracranial lipoma and a number of minor criteria of hemispheric atrophy, porencephalic cyst, calcification which was not basal ganglia calcification, alopecia, and frontotemporal subcutaneous lipoma.

*Diagnosis Based on Minor and Major Criteria*

*Definitive Case*

- Three systems involved with major criteria in 2 or more
- Three systems involved, proven NP or possible NP, and at least 1 minor skin criteria
- Two systems involved with major criteria, one of which is proven NP or possible NP and 1 or more minor skin criteria

*Probable Case*

- Two systems involved with major criteria
- Two systems involved, proven or possible NP.

## 2. Discussion

We present an unusual presentation of ECCL in a patient with no prior imaging for comparison. The syndrome is usually recognised and imaged at birth, but no such information was available in this overseas visitor. The patient presented with a skull fracture following a seizure, involving the moderately thinned, protuberant region of calvarium that had been remodelled by an underlying porencephalic cyst.

ECCL was first described in 1970 by Haberland and Perou [1, 2]. There is no specific genetic inheritance pattern and the condition occurs spontaneously with no gender predilection. To date, no cases in either siblings or children of affected individuals have been reported [2]. This neurocutaneous syndrome is a combination of central nervous system (CNS) and cutaneous and ocular abnormalities which can be unilateral or bilateral [2]. The aetiology of this disorder was suggested by Happle to be a lethal somatic mutation, able to survive due to a mosaic state close to other genetically normal cells [3]. Moog postulated the genetic aetiology to relate to mosaicism for a mutated autosomal gene involved in vasculogenesis and multiple mesenchymal tumours [2]. Recently, Bennett et al. have suggested that mosaic activating mutations in FGFR1 cause ECCL [4].

A broad spectrum of neurological impairments has been described, varying from normal to mild developmental delay at one end of a spectrum to profound development delay and psychomotor retardation on the other [2]. The presence and frequency of seizure activity also vary from no seizures in some patients, along a spectrum ending in severe protracted seizures or refractory epilepsy. There is no demonstrable correlation between the severity of neuroimaging findings and the severity of retardation or seizure frequency. Furthermore, imaging has yet to provide reliable prognostic features [5].

The cerebral anomalies in ECCL are postulated by Moog et al. to be mesenchymal abnormalities arising from neural crest derivatives, rather than primary cerebral malformations [5]. The commonest cerebral anomalies are lipomas which may be intracranial and/or intraspinal, and these are thought to result from abnormal differentiation and persistence of the meninx primitiva. The most common location for intracranial lipomas is at the cerebellopontine angle. Spinal lipomatosis is very commonly observed on spinal imaging and can extend for the entire length of the spinal cord [5].

A rare but reported association with anomalies of the corpus callosum varying from a thin corpus callosum to complete agenesis has been noted [5]. Asymmetric cerebral atrophy and unilateral cortical dysplasia are frequent imaging findings. The posterior fossa has also been reported to be affected in a small number of cases and include Dandy-Walker malformations and cerebellar hypoplasia and enlargement of the cerebellar cistern [5]. Intracranial calcifications are frequently seen but do not involve the basal ganglia. Rarely, intracranial blood vessels may be involved, including vascular abnormalities such as leptomeningeal angiomatosis or vascular malformations. Almost any combination of these malformations could be observed as there is incredible variability in this neurocutaneous disorder.

The cutaneous and ocular manifestations occur in a more consistent pattern than the CNS findings and are more commonly unilateral. They form an important part of revised diagnostic criteria proposed by Moog et al. (Table 1 and Diagnosis Based on Minor and Major Criteria). ECCL affects the dermis and hypodermis of the face and neck and the cutaneous hallmark is considered the presence of a nevus psiloliparus, a major skin criterion for the diagnosis of Haberland syndrome. This is a hairless fatty nevus which clinically looks like a bulging, soft scalp lesion with overlying alopecia which is usually nonscarring alopecia. Initially, it had been reported in the literature as a pathognomonic cutaneous manifestation of ECCL until Happle and Hörster described nevus psiloliparus nevus in two nonsyndromic cases [6]. Subcutaneous lipomas are common in the frontotemporal and zygomatic regions. Skin tags, nonscarring alopecia without a lipoma and focal areas of scalp aplasia or hypoplasia are minor cutaneous diagnostic criteria [2].

There are a number of important observed ocular anomalies. Benign ocular tumours such as choristomas are the commonest ophthalmologic manifestation [7] and a major diagnostic criterion. Ocular and eyelid colobomas, corneal anomalies, or abnormalities of the anterior chamber and calcification of the globe may occur [5]. Musculoskeletal system involvement with osteomas, odontomas, or ossifying fibromas of the jaw or multiple bone cysts may be present and aortic coarctation is the principal cardiovascular finding.

The differential diagnosis of ECCL includes several other neurocutaneous disorders with overlapping features. Proteus syndrome can also present with cutaneous lipomas and ocular choristomas. However, it has a more progressive course with asymmetric and disproportionate overgrowth of adipose tissues, soft tissues, connective tissue, or bone and can present with epidermal nevi, lipomas, or vascular malformations [7–9]. Oculocerebrocutaneous syndrome (Delleman Oorthuys syndrome) has some similar features but the most typical cutaneous manifestation is a supra-auricular hypoplastic skin defect as opposed to the nevus psiloliparus [2, 7, 10, 11]. Oculoectodermal syndrome (Toriello-Lacassie-Droste syndrome) does not involve intracranial lipomas and may be a forme fruste of ECCL [8, 12–14]. The family of epidermal naevus syndromes also overlap with ECCL but involve an alternate range of epidermal naevus manifestations to the typical nevus psiloliparus [2, 7].

Patients suffering skull fractures resulting from seizure-related injury should be considered for further evaluation for an underlying neuroparenchymal structural abnormality, ideally using MRI. Recognition of the characteristic neuroimaging and characteristic cutaneous manifestations of ECCL is crucial to establish this syndrome from its key differentials.

## Figures and Tables

**Table 1:** Diagnostic criteria (adapted from Moog et al.).

	Eye	Skin	CNS	Other
Major	Choristoma	Nevus psiloliparis (NP)	Intracranial or intraspinal lipoma	Jaw tumours:
				(i) Osteoma
				(ii) Odontoma
				(iii) Nonossifying fibroma
				Multiple bone cysts
				Aortic coarctation

Minor	Corneal anomalies	Possible NP	Abnormal intracranial vessels	
	Ant. chamber anomalies	Patchy/streaky nonscarring alopecia (without NP)	Arachnoid cyst	
			Abnormalities of meninges	
	Ocular coloboma	Frontotemporal subcutaneous lipomas	Hemispheric atrophy (complete or partial)	
	Eyelid coloboma	Focal aplasia/hypoplasia scalp	Porencephalic cyst(s)	
	Calcification of globe	Small nodular skin tags on outer eyelid (between outer canthus and tragus)	Asymmetrically dilated ventricles	
			Hydrocephalus	
			Calcification (not basal ganglia)	
